# Therapy and Antidepressant Use in 8- to 29-Year-Old Autistic Medicaid Enrollees With Depression

**DOI:** 10.1177/13623613261453106

**Published:** 2026-06-16

**Authors:** Meghan E. Carey, Laura Graham Holmes, Lindsay L. Shea, David S. Mandell, Diana Schendel, Brian K. Lee, Kristen Lyall

**Affiliations:** 1 Drexel University, Philadelphia, PA, USA; 2 University of Pennsylvania, Philadelphia, USA; 3 Hunter College, New York, USA

**Keywords:** autism, depression, health services, psychotherapy, antidepressant, Medicaid, adolescents, school-age children, transition-age youth, epidemiology

## Abstract

Promoting access to effective depression treatment represents a crucial opportunity to mitigate increased suicide risk. We examined treatment trajectories for 8- to 29-year-old autistic enrollees of Medicaid, the U.S. safety net insurance program, with a new claim for major depressive disorder (MDD; *N* = 44,074). Using group-based trajectory modeling, we identified groups with similar probabilities of receiving psychotherapy or antidepressants in the 5 months following their new MDD claim – a period of acute treatment need. We also examined odds of trajectory group membership by demographic factors and co-occurring intellectual disability (ID) using multinomial logistic regression. Trajectory modeling suggested existence of four treatment trajectories: no/limited treatment (39%), gradual treatment decline (21%), late treatment initiation (14%), and continuous treatment (25%). Adjusted odds of continuous treatment, relative to no/limited treatment, were lower for Black enrollees (odds ratio [OR]: 0.63, 95% confidence interval [CI]: [0.59, 0.68]), Hispanic enrollees (OR: 0.58, 95% CI: [0.54, 0.62]), and those with co-occurring ID (OR: 0.84, 95% CI: [0.79, 0.89]), and highest for 8- to 12-year-olds (OR: 1.80, 95% CI: [1.60, 2.03]) and females (OR: 1.15, 95% CI: [1.09, 1.21]). Many Medicaid-enrolled autistic people do not receive depression treatment. Improving treatment in autistic enrollees requires varied and multi-faceted approaches that must consider demographic and clinical factors.

**Lay Abstract:**

We wanted to know if autistic people on Medicaid get therapy or antidepressants after being diagnosed with depression. We also looked at whether people who are also diagnosed with intellectual disability, are female, are children, or are Black or Hispanic are more or less likely to get this care. Earlier studies show that just over half of the people on Medicaid get any treatment after being diagnosed with depression. But no one has looked closely at autistic people on Medicaid. Getting treatment can help reduce depression symptoms and may lower the risk of suicide. In our study, we looked at autistic people on Medicaid who were newly diagnosed with depression. We tracked whether they got therapy or antidepressants over the next 5 months. We grouped people based on how much treatment they got. The largest group (39%) got no treatment. The second group (21%) started treatment but did not continue for all 5 months. The third group (14%) started treatment around the third month and kept going for 2 months. The last group (25%) got treatment for all 5 months. Autistic people who were Black, Hispanic, or had intellectual disability were least likely to get enough treatment. Autistic children and females were most likely to get enough treatment. In short, many autistic people are not getting the care they need for depression. Some may not get enough treatment to feel better. This can make depression last longer and raise the risk of suicide. We need to improve access to care, especially for those who are least likely to get it.

## Introduction

Approximately one in five autistic people have co-occurring depression (Lai et al., 2019; Lugo-Marín et al., 2019). Evidence suggests less than half of neurotypical adolescents or adults receive treatment for their depression (Brody & Hughes, 2025; Flores et al., 2023), and autistic people may be less likely to receive depression treatment because of medical overshadowing (Maddox et al., 2018). Unmanaged depression contributes substantially to suicide (World Health Organization, 2017), the risk of which is 7-fold higher among autistic people than in the general population (Hedley & Uljarević, 2018; Hirvikoski et al., 2020). Promoting access to effective and appropriate depression treatment represents a crucial opportunity to mitigate increased suicide risk for autistic people.

Course of depression treatment depends on severity of presentation. Consequently, treatment approaches are individualized with no one-size-fits-all approach. Nonetheless, the American Academy of Child and Adolescent Psychiatrists (AACAP) publishes guidelines to advise treatment decisions (Walter et al., 2023). AACAP recommends depression management for children and adolescents *should* include psychotherapy and *could* include antidepressants, particularly selective serotonin reuptake inhibitors (SSRIs), as first-line treatment (hereafter, “guidance-indicated” treatment). Guidance on adult depression treatment from the American Psychological Association mirrors these recommendations (Guideline Development Panel for the Treatment of Depressive Disorders, 2019). While treatment plans are appropriately determined at the individual level, population-level studies of depression treatment are required to set mental health priorities and policies.

Medicaid is a safety net insurance program, and the largest payer of behavioral services in the United States, yet Medicaid-enrolled youth diagnosed with depression often do not receive guidance-indicated treatment (Cummings et al., 2019; Medicaid and CHIP Payment and Access Commission, 2015; Shea et al., 2022). Only 50% of Medicaid enrollees of ages 5–17 years received any psychotherapy or antidepressant prescriptions following their major depressive disorder (MDD) diagnosis in one study (Cummings et al., 2019). Prior studies of psychotropic medication use among Medicaid-enrolled autistic young people report high antidepressant use (20%–25%), but do not examine longitudinal treatment patterns or focus exclusively on autistic youth with depression (Mandell et al., 2008; Rast et al., 2023). Since Medicaid insures about one in three autistic people (Roux et al., 2023), this population represents an important group in which to examine gaps in depression treatment. Since prior research suggests that depression prevalence varies systematically by sex, race, and co-occurring intellectual disability (ID) among autistic people, which co-occurs in 40% of autistic individuals (Benevides et al., 2024; Lai et al., 2019; Rai et al., 2018; Shaw et al., 2025), there is potential for differences in treatment across intersecting identities.

Understanding of longitudinal depression service utilization among autistic people with MDD and the demographic and clinical factors influencing who engages in guidance-indicated treatment are necessary yet understudied. To address these gaps, we (a) identify different longitudinal patterns of treatment, or trajectory classes, for depression, including outpatient psychotherapy and pharmacotherapy, following an MDD claim among autistic Medicaid-enrolled youth, and (b) examine demographic and clinical factors that predict membership in different trajectory classes.

## Methods

### Study Design and Data Source

We conducted a retrospective cohort study using data from the Centers for Medicare and Medicaid Services (CMS). The data came from all 50 U.S. states and the District of Columbia between 2016 and 2019. We used Transformed Analytic Files (TAF) from Medicaid, which insures Americans near or below the poverty line, as well as those with qualifying disabilities (Medicaid.gov, n.d.; Rudowitz et al., 2023). The service (inpatient, outpatient, long-term services), prescription, and demographic and eligibility files available included data on healthcare claims, prescriptions, and demographic and enrollment characteristics, respectively.

### Sample

Our data extract included all enrollees, regardless of age, with an autism diagnosis. This was identified using the CMS Chronic Conditions Warehouse (CCW) algorithm (1 + inpatient or 2 + outpatient claims of F84.X) (Centers for Medicare & Medicaid Services, n.d.). The algorithm has been used in numerous studies of Medicaid-enrolled children and autistic adults (Bishop-Fitzpatrick & Rubenstein, 2019; Rast et al., 2023) and has an ~90% positive predictive value (Grosse et al., 2022). Autistic enrollees with MDD were also identified using 1 + inpatient or 2 + outpatient claims that included an F32 or F33 International Classification of Diseases, 10^th^ Revision, Clinical Modification (ICD-10-CM) code, consistent with literature and the CCW algorithm (Bishop-Fitzpatrick & Rubenstein, 2019; Zhang et al., 2024). Autism and MDD diagnostic codes and *Diagnostic and Statistical Manual of Mental Disorders*, Fifth Edition, (*DSM*-5) MDD conditions are provided in the Supplemental Material (Tables S1 and S2).

Supplemental Figures S1 and S2 detail inclusion criteria and an analytic timeline. Our initial sample included autistic Medicaid enrollees ages 8–29 years with MDD between 2016 and 2019 (*n* = 140,438). We selected this age range given evidence that (a) suicidal thoughts appear in autistic children as young as 8 (Schindel et al., 2024), and (b) that neurotypical depression onset is around the age of 25 years (National Institute of Mental Health, 2023). The first MDD claim during the study period was noted as their “index” claim (although they may have prior MDD claims not observable in our data).

We required 90 days of enrollment (3 months “washout”) before and 150 days (5 months “follow-up”) after their index claim (and that washout does not include time before 1 January 2016, and follow-up does not include time after 31 December 2019 where data were unavailable; *n* = 107,477 included, 23.5% excluded). For consistency with prior studies (Geier et al., 2015; Riehm et al., 2022), and to ensure complete longitudinal data, we then restricted to those with continuous enrollment during the washout and follow-up periods (defined as 8+ months enrollment, *n* = 84,025 included, 21.8% excluded). As we intended to include only those experiencing a new MDD episode, we also excluded those with any antidepressants prescribed during washout (*n* = 53,620 included, 36.2% excluded). Finally, we excluded those with private insurance (*n* = 44,074 included, 17.8% excluded). Because Medicaid is the payer of last resort (Medicaid and CHIP Payment and Access Commission, 2020), and we did not have access to private insurance data, this exclusion criterion ensures our ability to observe complete MDD treatment.

### Outcomes

Among those experiencing a new MDD episode, we wanted to examine their touchpoints with the mental healthcare system around the acute phase of their episode. The acute phase is generally the first 2–3 months after an MDD episode and is when a patient is in the highest need of depression treatment and risk of relapse (Moriarty et al., 2020). Consistent with the approach in Cummings et al. (2019), we also extended the follow-up period to 20 weeks (5 months). This allows some lead time while the patient navigates the mental healthcare system – finding an appropriate provider and filling their antidepressant prescription.

Our outcomes were developed based on receipt of guidance-indicated treatment for children, adolescents, and adults with MDD, which states that psychotherapy and pharmacotherapy via antidepressants are appropriate first-line treatments. Specifically, over the 5-month follow-up, we created indicators of whether an individual had any psychotherapy, and separately any pharmacotherapy, in each month. We identified psychotherapy visits using current procedure codes 90832-90834, 90836-90838, 90846, 90847, 90849, and 90853 in outpatient claims across outpatient settings (see Supplemental Table S3 for corresponding therapy structures) and counted those where depression was listed as a diagnosis on the claim. Pharmacotherapy included treatment with antidepressants identified in prescription data files based on drug classifications in the Multum drug database (Unified Medical Language System [UMLS], n.d.). The drug classifications included in analyses were antidepressants, SSRI antidepressants, miscellaneous antidepressants, phenylpiperazine antidepressants, tetracyclic antidepressants, Selective Serotonin and Norepinephrine Reuptake Inhibitors (SSNRI) antidepressants, tricyclic antidepressants, and monoamine oxidase inhibitors. We did not require that individuals have refills of the same antidepressant to be captured as having any pharmacotherapy that month.

#### Primary Outcome

Our outcome variable for primary analysis (group-based trajectory modeling, described below) collapsed the binary monthly indicators for whether an individual had received any psychotherapy (yes/no) or antidepressant prescription (yes/no) in each of the 5 months following their index MDD claim into an indicator of either treatment (yes/no any psychotherapy or antidepressant).

#### Secondary Outcomes

Secondary outcomes indicating receipt of depression services during follow-up were also categorized as (a) any psychotherapy and (b) minimally adequate psychotherapy (4+ visits within 5 months). Parallel outcomes were defined for pharmacotherapy (any antidepressant prescription, minimally adequate pharmacotherapy [3+ antidepressant prescriptions or refills within 5 months]) and receipt of either psychotherapy or pharmacotherapy. Definitions of minimally adequate treatment were adapted from prior work by Cummings et al. (2019), which incorporated AACAP guidelines, Healthcare Effectiveness Data and Information Set (HEDIS) measures, and prior research (Birmaher & Brent, 2007; National Quality Measures Clearing House, 2024; Stein et al., 2013). Specifically, AACAP suggests minimally 8–12 sessions of psychotherapy, and 84 days of pharmacotherapy when clinically indicated, following a major depression episode (Birmaher & Brent, 2007). Additional service measures (e.g. psychotherapy within 30 and 60 days) are provided in the Supplemental Material.

### Covariates

Covariates selected had *a priori* evidence of association with mental health service utilization and autism (Panchal & Lo, 2024; Shaw et al., 2025) and were grounded in the Andersen Behavioral Model of Health Services Use (Andersen & Newman, 1973). We hypothesized variation in treatment propensity by demographic factors – particularly developmental stage (age bands created for regression analyses for late childhood [8–12], adolescents [13–17], emerging adults [18–21], young adults [22–25], and adults [26–29]) and sex (male, female) (Cameron et al., 2021) – and co-occurring ID. Other demographic factors included race and ethnicity (American Indian and Alaska Native, Asian/Hawaiian/Pacific Islander, Black, Hispanic/Latino, Multiracial, White, Missing; included because of racial and ethnic differences in healthcare access and affordability (Chen et al., 2016)), Medicaid eligibility group (poverty, disability, other), and urbanicity (created following the U.S. Department of Agriculutre (USDA) Rural-urban Commuting Area Codes [RUCA]: urban, large town, small rural town, isolated small rural town; Economic Research Service, U.S. Department of Agriculture, 2023). Other co-occurring conditions were examined descriptively (anxiety and attention-deficit/hyperactivity disorder [ADHD]). All co-occurring conditions were identified using the same case definition as with autism and MDD (1 + inpatient or 2 + outpatient claims [Supplemental Table S1]).”

### Statistical Analysis

First, we examined descriptive statistics of the study population by demographic factors and co-occurring conditions. Then, we identified longitudinal trajectories of MDD treatment after the index MDD claim using group-based trajectory model (GBTM) analysis. GBTM is a longitudinal analytic approach that allows for identifying homogeneous clusters of individuals within a heterogeneous population, while describing the dynamic nature of health over time and as it relates to individual-level characteristics (Nagin, 1999). Specifically, individuals are clustered in groups based on having a similar predicted probability of the outcome. We used a two-stage model-selection process to create trajectory groups (Nagin, 2005). First, we tested unadjusted models to identify the most appropriate number of groups after deciding *a priori* to test 1–6 groups. Second, we determined the best polynomial function of the groups. This was accomplished by a comparison in Bayesian Information Criterion (BIC) scores, average posterior probabilities (>0.70), and odds of correct classification (>5.0; Supplemental Table S5). The most parsimonious polynomial function included all linear terms. The variability in average posterior probabilities for groups in our final model is provided in Supplemental Table S6. After determining our final model (based on group number and polynomial function, described in the Results), we then examined bivariate comparisons between each group and covariates. All covariates demonstrating associations in bivariate comparisons were included in the adjusted model. Finally, we conducted a multinomial logistic regression to predict odds of group membership by above-noted covariates (e.g. age, ID). Specifically, we used the groups defined by GBTM as outcomes in the regression models and estimated odds of group membership by covariates adjusted for one another.

### Sensitivity Analyses

To examine the potential impact of requiring continuous enrollment, we compared demographic factors and primary results to a sample with relaxed enrollment criteria defined by having more than 7 out of 8 months of enrollment. We also examined if trajectory groups would differ if, instead of our combined treatment outcome (psychotherapy OR pharmacotherapy), we modeled trajectories of psychotherapy and pharmacotherapy separately.

## Results

The demographic factors, co-occurring conditions, and therapeutic depression service touchpoints of the sample are provided in Table 1. The median age was 16 years (interquartile range [IQR]: 13–20); 24% had co-occurring ID; and 30% were female. Approximately half were non-White (46%), and the plurality (47%) were eligible for Medicaid due to disability. In the 5 months following their index MDD claim, 48% had 1 + depression psychotherapy, 33% had 1 + antidepressant prescription, and 64% had 1 + depression psychotherapy visit and/or 1 + antidepressant prescription.

**Table 1.** Demographic Characteristics, Co-Occurring Conditions, and Depression Treatment of 8- to 29-Year-Old Autistic Medicaid Enrollees With Continuous Enrollment and a New Major Depressive Disorder Claim Between 2016 and 2019.

Variables	Analytic sample (*N* = 44,074)	
**Demographics factors**		
** Age**, median (IQR)	16 (13, 20)	
	***n***	**%**
** Sex**		
Female	13,267	30.1
Male	30,807	69.9
** Race and ethnicity**		
American Indian and Alaska Native	561	1.3
Asian/Hawaiian/Pacific Islander	724	1.6
Black	6,508	14.8
Hispanic/Latino	7,664	17.4
Multiracial	344	0.8
White	23,901	54.2
Missing	4,372	9.9
** Eligibility group**		
Poverty	9,783	22.2
Disability	20,871	47.4
Other^a^	13,420	30.4
** Urbanicity**		
Isolated small rural town	1,281	2.9
Small rural town	2,098	4.8
Large rural city/Town	4,290	9.7
Urban	33,643	76.3
Missing	2,762	6.3
**Co-occurring conditions** ^b^		
** Intellectual disability**	10,632	24.1
** Attention-deficit/hyperactivity disorder (ADHD)**	26,009	59.0
** Anxiety**	26,858	60.9
**Depression treatment** ^c^		
** Any psychotherapy visit**	21,015	47.7
** Any antidepressant prescription**	14,585	33.1
** Any psychotherapy or antidepressant prescription**	28,074	63.7

a Medicaid TAF data include 76 total eligibility codes. Those not linked directly to poverty or disability were combined into this category and include groups such as: those with incomes >133% federal poverty line and under 65, expansion groups, and pregnant women.

b Those with 1 + inpatient or 2 + outpatient claims with appropriate ICD-10-CM codes between the enrollee’s first month with Medicaid enrollment until their last month of enrollment within available data (2016–2019).

c During the observed 150-day follow-up period.

**Figure 1.**
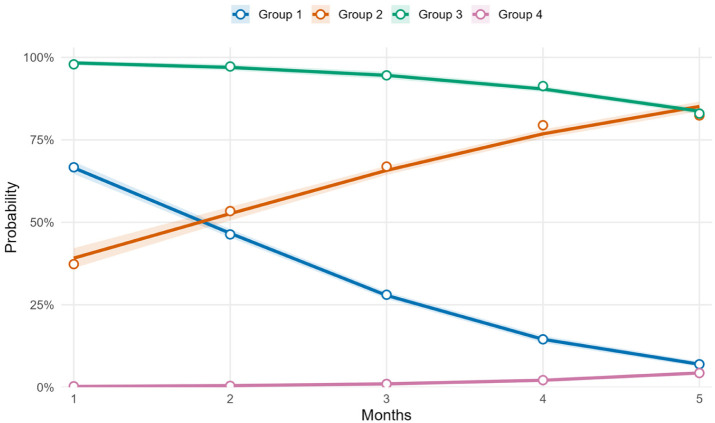
Group-based trajectory classes of treatment following the index major depressive disorder claim among 8- to 29-year-old autistic Medicaid enrollees, 2016–2019. Legend.

Results from Stage 1 model selection for GBTM are provided in the Supplemental Material (Table S4) along with the trajectory profile for each model (Supplemental Figure S3). Although there was BIC score improvement from the four- to five-group model (because lower BIC scores indicate better model fit), based on our visual inspection of the output, and the recommendation to maximize parsimony and interpretability (Nagin & Odgers, 2010), we selected the four-group model for analyses. Considering the four-group model (Figure 1), 39% of the sample had a near-zero probability of receiving any treatment (psychotherapy or pharmacotherapy) in the 5 months following diagnosis and were assigned to the “no/limited treatment” group (Group 4). Group 3, the next largest group (25%), included those with a very high probability of treatment throughout the 5-month follow-up (“continuous treatment” group). In the remaining sample, there were two distinct trajectories with opposite trends. Group 1 comprised 21% of our sample and contained individuals with a relatively high probability of treatment in the 30 days after their index MDD claim, but who had experienced a “gradual treatment decline” over the rest of follow-up. Group 2 comprised 14% of our sample and included those with “late treatment initiation.” Their probability of treatment increased from 40% to approximately 80% between 30 and 150 days after the index MDD claim.

**Table:** 

Group #	Group name	Proportion of sample
1	Gradual treatment decline	20.9%
2	Late treatment initiation	13.9%
3	Continuous treatment	25.0%
4	No/Limited treatment	39.1%

Table 2 provides the demographic characteristics of our sample by group. Compared to the other three trajectory groups, the continuous treatment group had a higher percentage of autistic people without ID (e.g. 80% vs. 71% of the no/limited treatment group) and lower percentage of Black and Hispanic autistic people (e.g. 15% Black vs. 20% of the no/limited treatment group). The continuous treatment group also had the lowest proportion of autistic individuals eligible for Medicaid because of disability (38% vs. 56% of the no/limited treatment group).

**Table 2.** Demographic Characteristics of 8- to 29-Year-Old Autistic Medicaid Enrollees Between 2016 and 2019, by Trajectory Groups of Major Depressive Disorder Treatment.

Variables	Gradual treatment decline (*n* = 9,208)	Late treatment initiation (*n* = 6,207)	Continuous treatment (*n* = 11,192)	No/limited treatment (*n* = 17,467)
**Age**, median (IQR)	16 (13, 20)	15 (13, 20)	15 (12, 19)	16 (13, 22)
**Intellectual disability** (%)				
Yes	21.4	22.2	20.1	28.8
No	78.6	77.8	79.9	71.2
**Sex** (%)				
Female	29.9	31.4	31.9	28.6
Male	70.1	68.6	68.1	71.4
**Race and ethnicity** (%)				
American Indian and Alaska Native	1.0	1.2	1.2	1.5
Asian/Hawaiian/Pacific Islander	1.5	1.5	1.6	1.8
Black	15.8	13.5	11.8	16.5
Hispanic/Latino	16.8	15.7	14.6	20.1
Multiracial	0.9	0.7	0.9	0.7
White	54.5	56.0	58.9	50.5
Missing	9.4	11.4	11.0	9.0
**Eligibility group**				
Poverty	23.5	23.1	25.1	19.3
Disability	44.6	44.6	37.8	55.9
Other^a^	31.9	32.3	37.1	24.8
**Urbanicity**				
Isolated small rural town	2.8	3.2	3.2	2.6
Small rural town	5.1	5.2	5.2	4.2
Large rural city/Town	10.2	9.8	9.9	9.4
Urban	75.9	75.3	74.6	78.1
Missing	6.0	6.6	7.2	5.7

a Medicaid TAF data include 76 total eligibility codes. Those not linked directly to poverty or disability were combined into this category and include groups such as: those with incomes >133% federal poverty line and under 65, expansion groups, and pregnant women.

**Figure 2.**
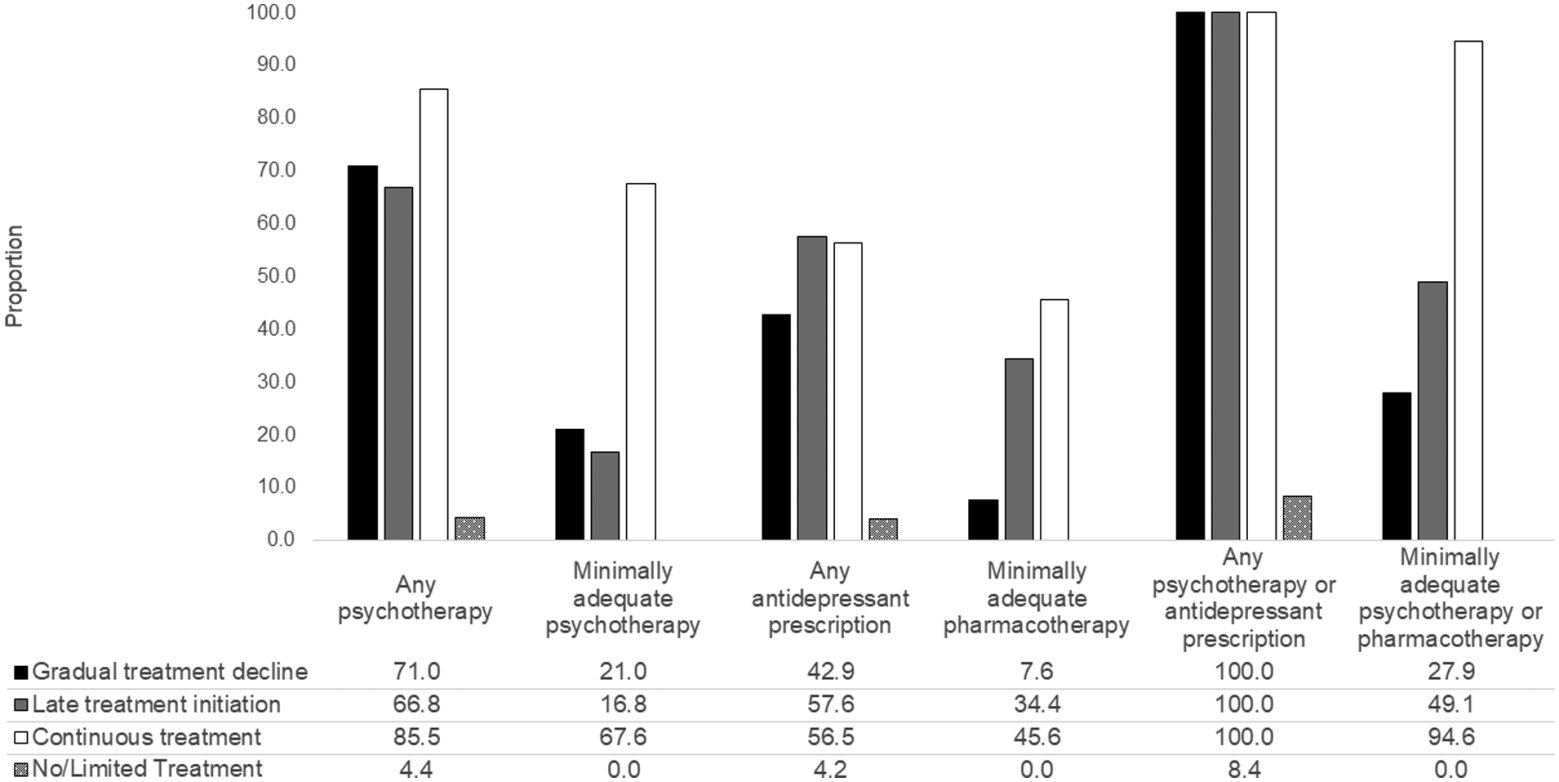
Proportion of 8- to 29-year-old autistic Medicaid enrollees receiving psychotherapy only, pharmacotherapy only, or either psychotherapy or pharmacotherapy during the 5 months following their index major depressive disorder claim during 2016–2019, by trajectory group.

The proportion of each group receiving psychotherapy only, pharmacotherapy only, or either treatment modality in the 5-month follow-up by trajectory group is presented in Figure 2. Overall, those in the continuous treatment group had the highest proportions achieving minimally adequate psychotherapy (67.6%) or pharmacotherapy (45.6%). A higher proportion of those with gradual treatment decline had minimally adequate psychotherapy (21.0%) compared to pharmacotherapy (7.6%). We observed the opposite relationship in the late treatment initiation group: 16.8% had minimally adequate psychotherapy, and 34.4% had pharmacotherapy. Overall, the proportion achieving minimally adequate psychotherapy or pharmacotherapy was low across groups. Additional related measures of depression service utilization are provided in the Supplemental Material (e.g. first psychotherapy within 30 days, Supplemental Table S7).

The crude and adjusted ORs of group membership by demographic characteristics and co-occurring ID are provided in Table 3. Estimates from the adjusted model were generally consistent with those from crude models in association with strength and direction though the magnitude was commonly attenuated in adjusted models. After adjusting for age group, sex, race and ethnicity, and Medicaid eligibility group, autistic young people with ID had 16% lower odds of belonging to the continuous treatment group (referent group: no/limited treatment group) than those without ID. The late treatment initiation and gradual treatment decline groups were similar in the odds of group membership among individuals with autism and co-occurring ID (e.g. late treatment initiation adjusted OR: 0.79, 95% CI: [0.74, 0.85]). Compared to 26- to 29-year-olds, the youngest age groups had increased odds of belonging to the continuous treatment group (8- to 12-year-olds adjusted OR: 1.80, 95% CI: [1.60, 2.03]) relative to the no/limited treatment group. A similar pattern was found for other treatment groups by age group such that the youngest age groups had increased odds of group membership compared to 26- to 29-year-olds (gradual treatment decline adjusted OR: 1.57, 95% CI: [1.36, 1.81]). Black and Hispanic Medicaid enrollees had lower odds of being in any of the groups receiving treatment than of being in the no/limited treatment group. Compared to White individuals, Hispanic and Latino individuals had 42% lower odds of being in the continuous treatment group (OR: 0.58, 95% CI: [0.54, 0.62] relative to no/limited group) and 34% lower odds of being in the gradual treatment decline group (OR: 0.66, 95% CI: [0.61, 0.72]). Compared to individuals who were Medicaid-eligible due to poverty, those eligible due to disability had 42% lower odds of belonging to the continuous treatment group (OR: 0.58, 95% CI: [0.54, 0.62]), and other treatment groups showed similar associations (e.g. gradual treatment decline OR: 0.72, 95% CI: [0.67, 0.78]).

**Table 3.** Adjusted Odds of Membership to Each Trajectory Group Relative to the No/Limited Treatment Group by Demographic and Diagnostic Characteristics of Medicaid Enrollees of Ages 8–29, 2016–2019.

	Late treatment initiation vs. no/limited		Gradual treatment decline vs. no/limited		Continuous treatment vs. no/limited	
Variables	Crude OR [95% CI]	Adj. OR^a^ [95% CI]	Crude OR [95% CI]	Adj. OR^a^ [95% CI]	Crude OR [95% CI]	Adj. OR^a^ [95% CI]
**Intellectual disability**						
Yes	0.67 [0.64, 0.72]	0.79 [0.74, 0.85]	0.70 [0.66, 0.75]	0.85 [0.79, 0.91]	0.62 [0.59, 0.66]	0.84 [0.79, 0.89]
No	**Ref**	**Ref**	**Ref**	**Ref**	**Ref**	**Ref**
**Age group**						
8–12	1.70 [1.51, 1.92]	1.54 [1.35, 1.74]	1.67 [1.45, 1.92]	1.57 [1.36, 1.81]	2.05 [1.83, 2.30]	1.80 [1.60, 2.03]
13–17	1.72 [1.52, 1.95]	1.58 [1.40, 1.79]	1.67 [1.45, 1.92]	1.58 [1.37, 1.82]	1.85 [1.64, 2.08]	1.65 [1.46, 1.85]
18–21	1.28 [1.12, 1.46]	1.26 [1.11, 1.44]	1.14 [0.98, 1.33]	1.13 [0.97, 1.32]	1.10 [0.97, 1.24]	1.07 [0.95, 1.22]
22–25	1.04 [0.91, 1.20]	1.07 [0.94, 1.23]	1.00 [0.85, 1.17]	1.03 [0.88, 1.20]	0.95 [0.83, 1.08]	0.99 [0.87, 1.13]
26–29	**Ref**	**Ref**	**Ref**	**Ref**	**Ref**	**Ref**
**Sex**						
Female	1.06 [1.00, 1.12]	1.05 [1.00, 1.11]	1.14 [1.07, 1.21]	1.13 [1.06, 1.21]	1.17 [1.11, 1.23]	1.15 [1.09, 1.21]
Male	**Ref**	**Ref**	**Ref**	**Ref**	**Ref**	**Ref**
**Race and ethnicity**						
American Indian and Alaska Native	0.65 [0.51, 0.82]	0.62 [0.49, 0.78]	0.71 [0.54, 0.92]	0.67 [0.51, 0.87]	0.69 [0.56, 0.85]	0.64 [0.51, 0.79]
Asian/Hawaiian/Pacific Islander	0.82 [0.67, 1.00]	0.79 [0.64, 0.96]	0.79 [0.62, 0.99]	0.76 [0.60, 0.96]	0.79 [0.66, 0.95]	0.75 [0.62, 0.91]
Black	0.89 [0.82, 0.95]	0.91 [0.85, 0.98]	0.74 [0.68, 0.81]	0.75 [0.69, 0.82]	0.61 [0.57, 0.66]	0.63 [0.59, 0.68]
Hispanic/Latino	0.78 [0.72, 0.83]	0.74 [0.69, 0.79]	0.70 [0.65, 0.76]	0.66 [0.61, 0.72]	0.62 [0.58, 0.67]	0.58 [0.54, 0.62]
Multiracial	1.16 [0.87, 1.54]	1.19 [0.89, 1.60]	0.86 [0.60, 1.23]	0.88 [0.61, 1.25]	1.13 [0.86, 1.47]	1.19 [0.91, 1.56]
Missing	0.97 [0.89, 1.06]	1.01 [0.92, 1.10]	1.14 [1.04, 1.26]	1.19 [1.07, 1.31]	1.05 [0.97, 1.14]	1.12 [1.03, 1.22]
White	**Ref**	**Ref**	**Ref**	**Ref**	**Ref**	**Ref**
**Eligibility group**						
Disability	0.65 [0.61, 0.70]	0.72 [0.67, 0.77]	0.67 [0.62, 0.72]	0.72 [0.67, 0.78]	0.52 [0.49, 0.56]	0.58 [0.54, 0.62]
Other^b^	1.06 [0.98, 1.14]	0.98 [0.91, 1.05]	1.09 [1.00, 1.18]	0.97 [0.90, 1.06]	1.15 [1.08, 1.23]	1.00 [0.93, 1.07]
Poverty	**Ref**	**Ref**	**Ref**	**Ref**	**Ref**	**Ref**

*Note.* Adj. = adjusted; OR = odds ratio; CI = confidence interval; Ref = referent category; TAF = Transformed Analytic Files.

a Adjusted for all other variables included in the table.

b Medicaid TAF data include 76 total eligibility codes. Those not linked directly to poverty or disability were combined into this category and include groups such as: those with incomes >133% federal poverty line and under 65, expansion groups, and pregnant women.

To test the impact of our continuous enrollment inclusion criteria on results, we conducted a sensitivity analysis with participants with up to 1 month of disenrollment (required 7 out of 8 months enrollment; “disenrollment sample”). Participant demographic characteristics were comparable to the primary analytic sample for all covariates (Supplemental Table S8). We also compared treatment use between the primary analytic sample and the disenrollment sample (Supplemental Table S9), and results did not substantively change. When examining treatment trajectories of psychotherapy (Supplemental Figure S4) and pharmacotherapy (Supplemental Figure S5), we concluded that (a) for each treatment modality, we would still choose the four-group model, and (b) that treatment trajectories looked relatively consistent with the primary analysis using psychotherapy and pharmacotherapy combined.

## Discussion

While studies have assessed trajectories of depressive symptoms over time in autistic people (Tieu et al., 2022; Ulvenes et al., 2022), this is the first known study to examine treatment of MDD in young autistic Medicaid beneficiaries at the population level. Using group-based trajectory modeling, we found that 39% of 8- to 29-year-old autistic Medicaid beneficiaries received no/limited treatment in the 5 months following a new claim for MDD. The remaining 60% of our sample fell into one of three treatment groups: (a) “continuous” treatment (25%); (b) gradual treatment decline (21%); and (c) late treatment initiation (14%). Mental health treatment broadly is known to have life-saving impact among autistic people (Camm-Crosbie et al., 2019). However, even among those who did receive some treatment, the majority of autistic Medicaid enrollees did not receive minimally adequate treatment for MDD during the period of highest acuity.

Our largest treatment trajectory group (39%) consisted of autistic Medicaid enrollees who had either no or extremely limited treatment in the 5 months following their first MDD claim. Compared to a general sample of Medicaid enrollees, autistic Medicaid enrollees were 2.5 times as likely to receive no treatment (16.4% of beneficiaries of ages 5–17 in prior study; Cummings et al., 2019). One explanation is that system-level factors, such as limited trained professionals who accept Medicaid insurance and/or are adequately trained to support autistic people, exist (Adams & Young, 2021; Maddox et al., 2019). Relatedly, autistic beneficiaries who are Medicaid-enrolled through disability waivers may more easily access other behavioral health supports covered by intellectual and development disability services, such as assertive community treatment (Peebles & Bohl, 2014). Our findings support this hypothesis because we observed that individuals enrolled in Medicaid through disability waivers were less likely to be in the continuous treatment group than those enrolled in Medicaid because of limited fiscal resources. The appropriateness of alternative services in place of guidance-indicated treatment warrants further inquiry. Future research in this area with an understanding of the preferences of the autistic community is needed.

We also found that 25% of the sample received “continuous treatment” following their index MDD claim. Compared to the group with “no/limited treatment,” those with continuous treatment were more likely to not have co-occurring ID, be younger, and be White. The younger group having higher odds of being in the continuous treatment group likely speaks to (a) familial advocacy and facilitation for treatment (Brown et al., 2016; Han et al., 2017) or (b) depression severity (Magaard et al., 2017). Relatedly, our findings substantiate the argument of a “services cliff” that autistic people face, which include challenges with healthcare navigation during emerging adulthood (Betz et al., 2013), including in the Medicaid system (Carey et al., 2023). These findings suggest that emerging adults would particularly benefit from interventions to improve service navigation for depression treatment. Our result that young Black and Hispanic enrollees were less likely to receive depression treatment than White enrollees is consistent with prior work (Cummings et al., 2019; Flores et al., 2023) and reiterates the need for strategies to address disparities in navigating health systems.

Two other treatment trajectories emerged, suggesting a varied experience within the Medicaid system. The group that experienced “gradual treatment decline” had a 70% probability of receiving psychotherapy or pharmacotherapy 30 days after the index MDD claim, but the probability of care at 3 and 5 months was around 30% and 10%, respectively. Two possible explanations for these experiences include that group membership could reflect (a) those who have mild/moderate depression and reach remission quickly and appropriately terminate services; or (b) dissatisfaction with treatments or providers. To the latter point, the inability of mental health providers to adapt psychotherapy for autistic needs (Brede et al., 2022) and adverse effects of antidepressant treatment (Strawn et al., 2023) may lead to early discontinuation of treatment.

The group with “late treatment initiation” included individuals whose probability of services went from less than 40% at 30 days after the index MDD claim to 80% at 5 months after their index claim. This group likely reflects individuals who struggled to find therapists accepting autistic patients, and those who were placed on long waitlists prior to being seen, which has been consistently reported in research and systematic reviews (Adams & Young, 2021).

Key strengths of this work include its large sample size and focus on subpopulations within autism, its ability to fill gaps in our understanding of MDD treatment of autistic people, and the novel use of group-based trajectory modeling to unearth heterogeneity in treatment trajectories. Furthermore, Medicaid is the largest payer of behavioral health services in the United States (Medicaid and CHIP Payment and Access Commission, 2015), making it a highly relevant dataset in which to examine treatment trajectories within autism. Our sample of autistic Medicaid enrollees is highly diverse, with 46% being non-White, including 15% Black and 17% Hispanic autistic individuals. These proportions represent a two-fold increase in Black and Hispanic representation compared with averages reported in systematic reviews of autism research samples (8% and 9% of samples, respectively) (Steinbrenner et al., 2022). As a result, the present findings are well positioned to inform equitable policy and service delivery decisions for diverse autistic populations.

Study limitations should also be considered. First, Medicaid claims have no measure of depression severity, which may impact group membership. For example, if youth with severe depression are more likely to seek care than those with mild/moderate depression (Magaard et al., 2017), they might comprise more of the continuous treatment than the no/limited treatment group. Relatedly, identification of depression relies on diagnoses from providers. Providers rely on the screening and diagnostic tools available. While many depression-screening tools are validated among school-age children and adolescents (Johnson et al., 2002), we acknowledge that these tools imperfectly assess depression in autistic youth. Second, although we required autism diagnosis to precede depression claims, and most of our sample was <16 years of age at the time of depression, we did not have information on age at autism diagnosis. We therefore could not assess the impact of age at diagnosis, which may influence depression recognition and treatment (Jadav & Bal, 2022). Third, we cannot rule out measurement error in our treatment categories by participants changing antidepressant medication or those who had a 90-day initial prescription (the latter is rare when initiating antidepressants). Relatedly, our analysis also did not examine type of medications used, which may have impacted trajectory group findings, particularly if different medications cause side effects that impact future treatments (Strawn et al., 2023), and future work should address this possibility with detailed information on trajectories of medication use types over time. Fourth, data selection methods and/or study inclusion criteria may have introduced selection bias (Reilly et al., 2005). We did address this by examining demographic factors by those with and without continuous enrollment, as well as by depression treatment trajectories, which suggested comparability in the samples. Fifth, we cannot rule out the potential for unmeasured confounding by factors not available in claims data (e.g. education level). However, a prior claims study found that unlike in the general population, education and other social determinants of health may not impact the propensity of autistic individuals to seek mental health treatment (Smith et al., 2025). Sixth, we did not examine how mental health conditions co-occurring with autism and depression affected pharmacotherapy or psychotherapy patterns. This could affect an individual’s probability to receive depression treatment if, for example, they are on another medication that cannot be taken in combination with antidepressants. Finally, although group-based trajectory modeling is inherently subjective, the four-group model was selected because the alternative five-group model offered only marginal improvement in fit and introduced a duplicative small group also characterized by gradual treatment decline. Prioritizing clarity and policy relevance, we chose the more parsimonious model.

## Conclusions

Among our national sample of 8- to 29-year-old autistic Medicaid beneficiaries, we found that treatment for MDD in the acute phase, for most, is not minimally adequate. For autistic Medicaid enrollees, those who were Black or Hispanic, those with co-occurring ID, and those in “emerging adult” age groups may be least likely to receive minimally adequate treatment for MDD, which should be considered in outreach and intervention efforts. Our results suggest that improving MDD treatment trajectories for autistic people will require efforts to both improve timely access to and quality of services.

## Supplemental Material

sj-docx-1-aut-10.1177_13623613261453106 – Supplemental material for Therapy and Antidepressant Use in 8- to 29-Year-Old Autistic Medicaid Enrollees With Depression Supplemental material, sj-docx-1-aut-10.1177_13623613261453106 for Therapy and Antidepressant Use in 8- to 29-Year-Old Autistic Medicaid Enrollees With Depression by Meghan E. Carey, Laura Graham Holmes, Lindsay L. Shea, David S. Mandell, Diana Schendel, Brian K. Lee and Kristen Lyall in Autism
