# Supplementary material for: Therapy and Antidepressant Use in 8- to 29-Year-Old Autistic Medicaid Enrollees With Depression
Source: Autism. 2026 Jun 16;30(8):2025–37. doi: 10.1177/13623613261453106 (PMC13278382; doi:10.1177/13623613261453106)
Supplement: sj-docx-1-aut-10.1177_13623613261453106 – Supplemental material for Therapy and Antidepressant Use in 8- to 29-Year-Old Autistic Medicaid Enrollees With Depression [file sj-docx-1-aut-10.1177_13623613261453106.docx]

| **Table S1**. Conditions of interest and diagnostic codes for case determination | |
| --- | --- |
|  | **ICD-10-CM codes** |
| **Autism** | F84.X |
| **Major Depression** | F32.0, F32.1, F32.2, F32.3, F32.4, F32.5, F32.89, F32.9, F32.A, F33.XX |
| **Intellectual Disability** | E78.71, E78.72, F70.XX, P04.3, Q86.0, Q87.1X, Q87.2, Q87.3, Q87.5, Q87.81, Q87.89, Q89.7, Q89.8, Q90.X, Q91.X, Q92.XX, Q93.XX (excluding Q93.82), Q95.2, Q95.3, Q99.2 |
| **Attention-deficit/hyperactivity disorder** | F63.0, F63.1, F63.2, F63.3, F63..81, F6389, F63.9, F90.0, F90.1, F90.2, F90.8, F90.9, F91.0, F91.1, F91.2, F91.3, F91.8, F91.9 |
| **Anxiety** | F06.4, F40.00, F40.01, F40.02, F40.10, F40.11, F40.210, F40.218, F40.220, F40.228, F40.230, F40.231, F40.232, F40.233, F40.240, F40.241, F40.242, F40.243, F40.248, F40.290, F40.291, F40.298, F40.8, F40.9, F41.0, F41.1, F41.3, F41.8, F41.9, F42, F42.2, F42.3, F42.4, F42.8, F4.29, F43.0, F43.10, F43.11, F43.12, F44.9, F45.8, F48.8, F48.9, F93.8, F99, R45.2, R45.5, R456, R457 |

| **Table S2.** ICD-10-CM codes for major depression and their DSM-5 counterpart | |
| --- | --- |
| **ICD-10-CM code(s)** | **DSM-5 condition** |
| F32.0 | Major depressive disorder, single episode, mild |
| F32.1 | Major depressive disorder, single episode, moderate |
| F32.2 | Major depressive disorder, single episode, severe without psychotic features |
| F32.3 | Major depressive disorder, single episode, severe with psychotic features |
| F32.4 | Major depressive disorder, single episode, in partial remission |
| F32.5 | Major depressive disorder, single episode, in full remission |
| F32.89 | Other specified depressive episodes |
| F32.9 | Major depressive disorder, single episode, unspecified |
| F32.A | Depression, unspecified |
| F33.XX | Major depressive disorder, recurrent |

| **Figure S1.** Flowchart of study inclusion and exclusion criteria |
| --- |
|  |
| ^a^ We required individuals be completely observable for 90 days prior to their index claim and 150 days after. Since we did not have data before January 1, 2016 or after December 31, 2019 any individuals with washout or treatment periods extending beyond this time were not completely observable in our data and thus removed from analyses.  ^b^ Medicaid enrollment files provided indicators of whether or not individuals were enrolled for each given month. We extracted month information from the first day of the washout period and last day of the treatment period, and required that individuals be enrolled all months from the first month of washout through the last month of follow-up.  ^c^ Adaptations to the Cummings et al (2019) inclusion/exclusion criteria were made for this criteria: 1) due to missing data issues, prescription date rather than fill date was used to identify antidepressant prescriptions; and 2) we indexed on first MDD claim, there were no MDD claims during the 90-day washout period, and thus that criterion was not applied to our sample  ^d^ Indication of third-party liability insurance or other coverage on any claim from Medicaid eligibility files was used to create the private insurance indicator variable |

| **Figure S2.** Example timeline for analytic sample |
| --- |
| 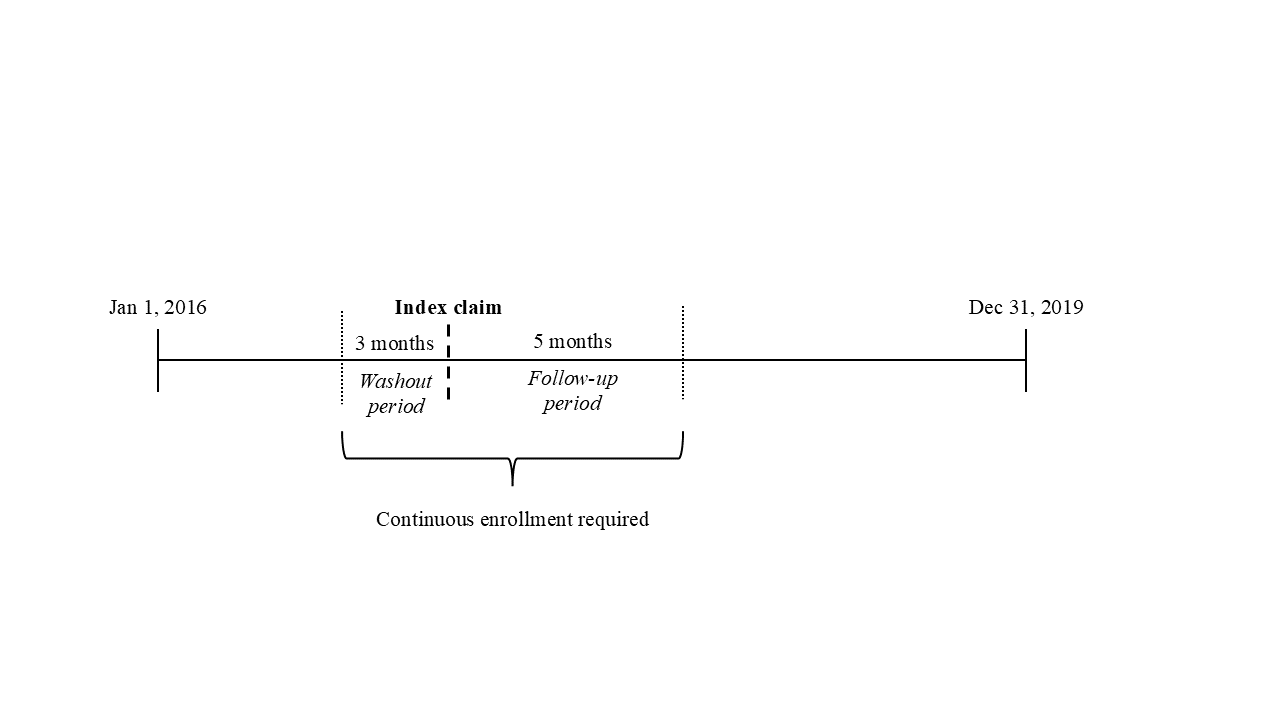 |

| **Table S3.** Current Procedural Terminology Codes for Psychotherapy Visits | |
| --- | --- |
| Individual Psychotherapy | 90832-90834, 90836-90838 |
| Family Psychotherapy | 90846, 90847, 90849 |
| Group Psychotherapy | 90853 |

| **Table S4**. Stage 1 model selection: Selecting group numbers by comparing model fit using Bayesian Information Criterion (BIC) and number of groups with <5% of the sample | | | | |
| --- | --- | --- | --- | --- |
| **# groups** | **Polynomial order^a^** | **BIC**  **(N = 44,074)^b^** | **BIC**  **(N = 220,370)^c^** | **N < 5%** |
| 1 | 1 | -148,935.3 | -148,936.9 | 0 |
| 2 | 1, 1 | -113,206.9 | -113,210.9 | 0 |
| 3 | 1, 1, 1 | -111,142.1 | -111,148.6 | 0 |
| 4 | 1, 1, 1, 1 | -109,848.4 | -109,857.3 | 0 |
| 5 | 1, 1, 1, 1, 1 | -109,525.2 | -109,536.4 | 0 |
| 6 | 1, 1, 1, 1, 1, 1 | -109,468.8 | -109,482.5 | 2 |
| ^a^ It’s recommended when there is no a priori understanding of polynomial order, to test all groups with the linear function for the first-stage of model selection  ^b^ Number of individuals in the analytic sample  ^c^ Total number of data (time points * N) | | | | |

| **Figure S3.** Testing combined trajectory group numbers from 1-6 |
| --- |

| 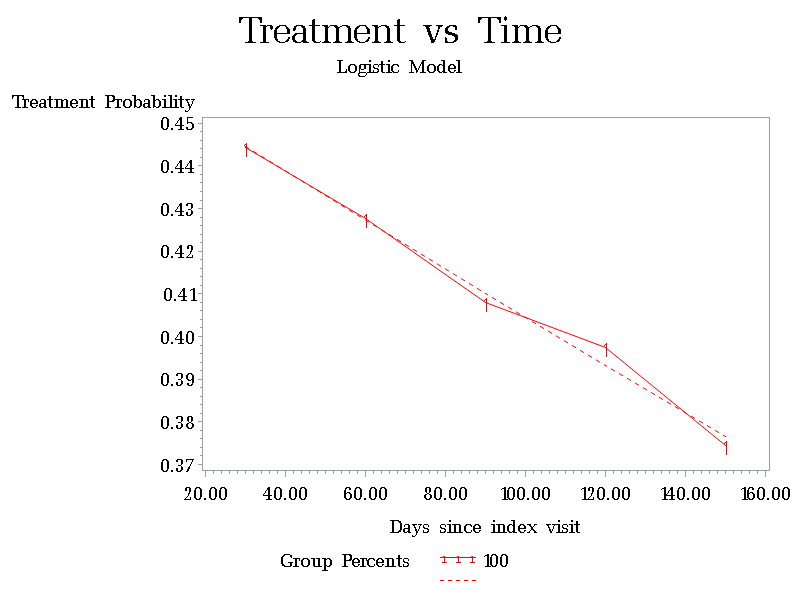 | 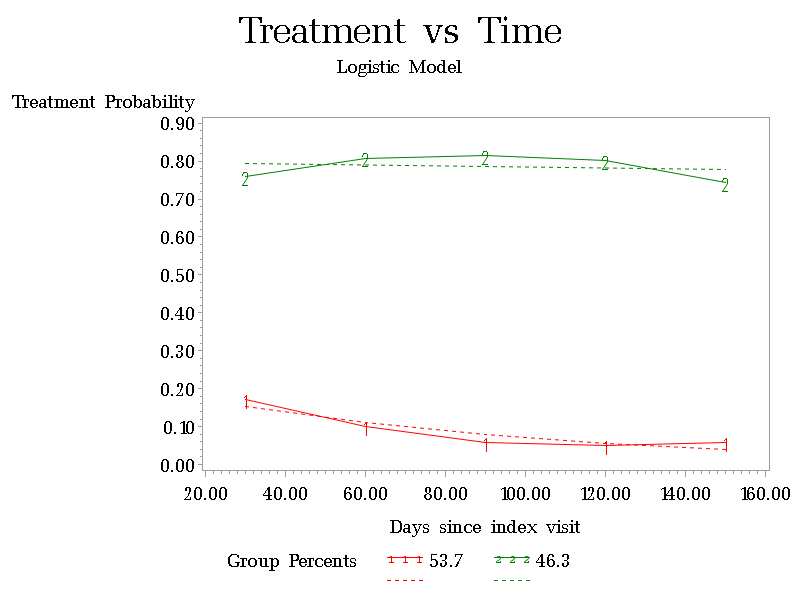 |
| --- | --- |
| 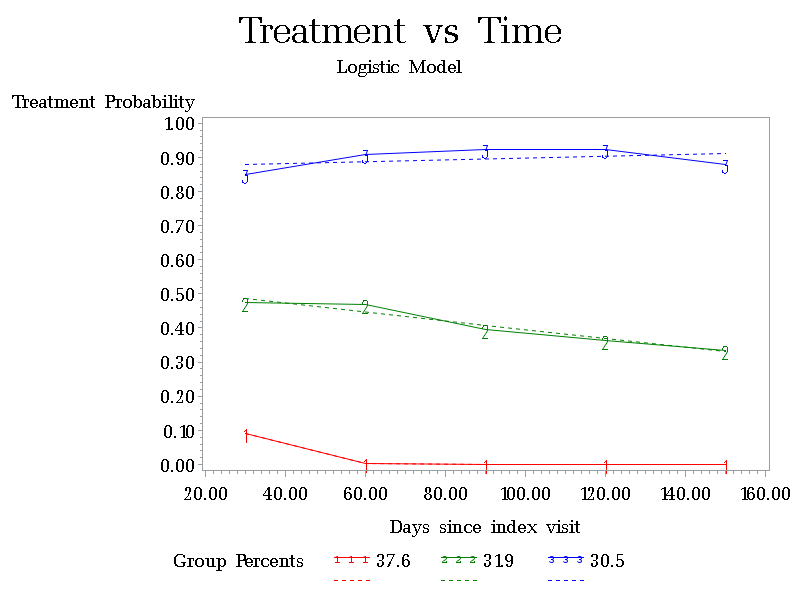 | 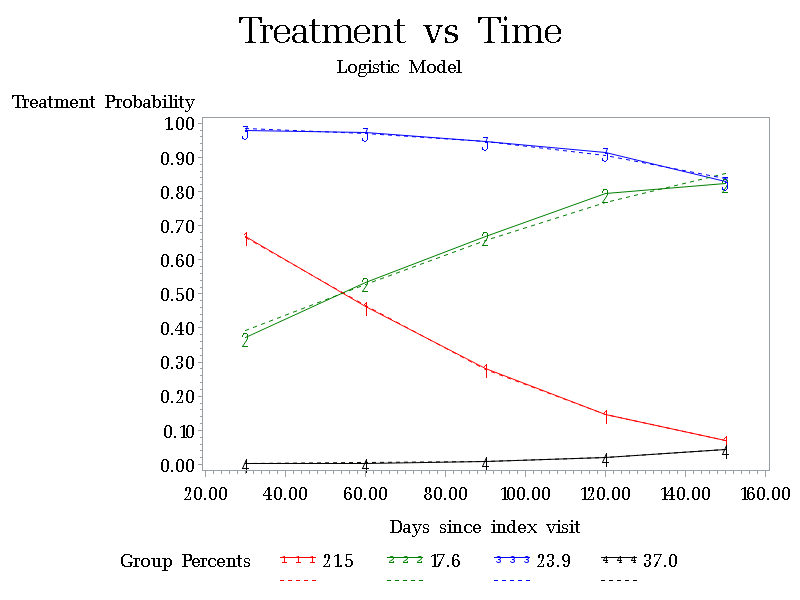 |
| 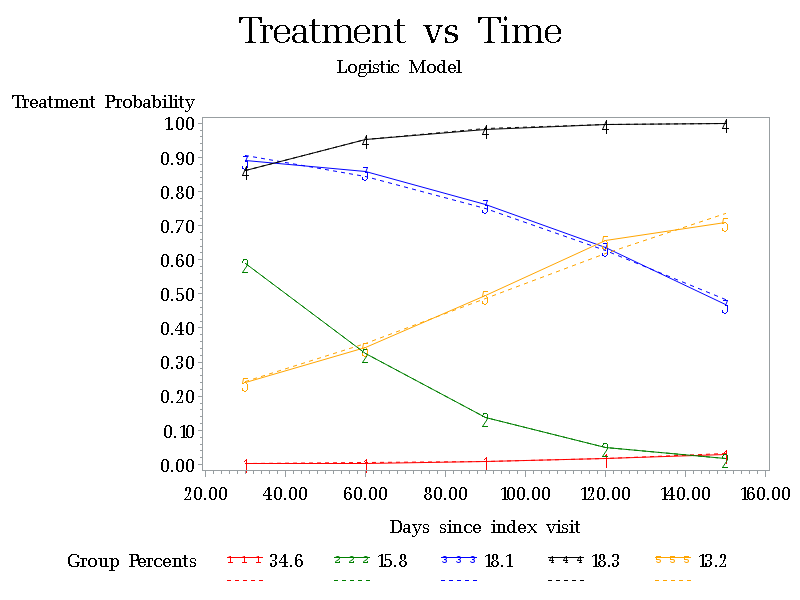 | 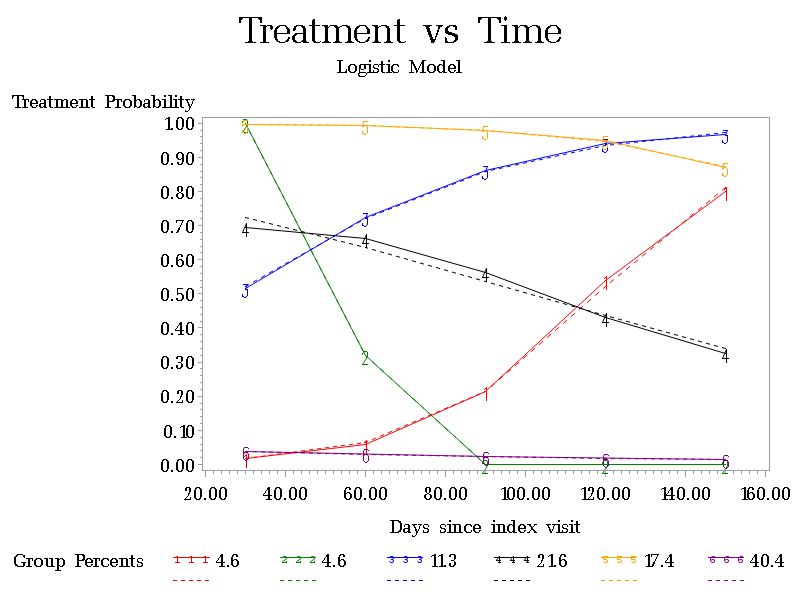 |

| **Table S5**. Stage 2 model selection: Selecting 5-group polynomial order by comparing model fit using Bayesian Information Criterion (BIC) and number of groups with <5% of the sample | | | | | | | | | | | | |
| --- | --- | --- | --- | --- | --- | --- | --- | --- | --- | --- | --- | --- |
|  |  |  |  |  | **AvePP^d^** | | | | **OCC^e^** | | | |
| **Model #** | **Polynomial order^a^** | **BIC**  **(N = 44,074)^b^** | **BIC**  **(N = 220,370)^c^** | **N < 5%** | **G1** | **G2** | **G3** | **G4** | **G1** | **G2** | **G3** | **G4** |
| **4-group model** | | | | | | | | | | | | |
| 4.0 | 1,1,1,1 | -109,848.4 | -109,857.3 | 0 | 0.84 | 0.86 | 0.86 | 0.92 | 19.2 | 28.8 | 19.6 | 19.6 |
| 4.1 | 2,2,2,2 | -109,454.3 | -109,466.3 | 0 | 0.79 | 0.79 | 0.89 | 0.89 | 13.7 | 17.6 | 25.8 | 13.8 |
| 4.2 | 2,2,1,2 | -109,561.2 | -109,572.5 | 0 | 0.91 | 0.82 | 0.88 | 0.76 | 36.9 | 21.3 | 23.4 | 5.4 |
| 4.3 | 2,3,1,2 | -109,454.3 | -109,466.3 | 0 | 0.89 | 0.89 | 0.77 | 0.81 | 29.5 | 37.9 | 10.7 | 7.3 |
| ^a^ It’s recommended when there is no a priori understanding of polynomial order, to test all groups with the linear function for the first-stage of model selection, then increase to highest order suspected of groups. Insignificant p-values from parameter estimates of higher order polynomials was used as evidence to remove higher order polynomial functions  ^b^ Number of individuals in the analytic sample  ^c^ Total number of data (time points * N)  ^d^ Nagin (2005) suggests a good fitting model exists all groups have average posterior probabilities (AvePP) of >0.7  ^e^ Nagin (2005) suggests a good fitting model exists when all groups have odds of correct classification (OCC) of >5.0, and larger OCC is better | | | | | | | | | | | | |

| **Table S6.** Variability in average posterior probabilities by group | | | |
| --- | --- | --- | --- |
| **Group #** | **Average Posterior Probability** | **Min** | **Max** |
| 1 | 0.84 | 0.45 | 0.94 |
| 2 | 0.86 | 0.70 | 0.99 |
| 3 | 0.86 | 0.52 | 0.92 |
| 4 | 0.92 | 0.56 | 0.94 |

| **Table S7**. Proportion receiving psychotherapy only, pharmacotherapy only, or either during the five months following their index major depressive disorder claim among autistic Medicaid-enrollees 8-29 years during 2016-2019, by trajectory group | | | | | |  |
| --- | --- | --- | --- | --- | --- | --- |
|  | **Gradual treatment decline**  (n = 9,208) | **Late treatment initiation**  (n = 6,207) | **Continuous treatment**  (n = 11,192) | **No/Limited Treatment**  (n = 17,467) | | |
| ***Psychotherapy*^a^** (%) |  |  |  |  | | |
| Any psychotherapy visit^a^ | 71.0 | 66.8 | 85.5 | 4.4 | | |
| First Psychotherapy within 30 days | 53.7 | 12.6 | 76.4 | 0.0 | | |
| First Psychotherapy within 60 days | 65.6 | 39.2 | 81.5 | 0.0 | | |
| Psychotherapy visit at all 5 timepoints^a^ | 0.0 | 0.0 | 46.0 | 0.0 | | |
| Minimally adequate  psychotherapy (> 4 visits)^b^ | 21.0 | 16.8 | 67.6 | 0.0 | | |
| > 8 psychotherapy visits^c^ | 5.7 | 3.2 | 35.4 | 0.0 | | |
| ***Pharmacotherapy*** (%) |  |  |  |  | | |
| Any antidepressant medication prescription^a^ | 42.9 | 57.6 | 56.5 | 4.2 | | |
| First antidepressant within 30 days | 29.8 | 9.9 | 42.4 | 0.0 | | |
| First antidepressant within 60 days | 38.0 | 31.2 | 49.4 | 0.0 | | |
| Antidepressant prescription at all 5 timepoints^a^ | 0.0 | 0.0 | 20.2 | 0.0 | | |
| Minimally adequate pharmacotherapy^d^ | 7.6 | 34.4 | 45.6 | 0.0 | | |
| ***Either psycho- or pharmacotherapy (%)*** |  |  |  |  | | |
| Any psychotherapy or pharmacotherapy^a^ | 100.0 | 100.0 | 100.0 | 8.4 | | |
| First psychotherapy or pharmacotherapy within 30 days | 77.1 | 20.8 | 100.0 | 0.0 | | |
| First psychotherapy or pharmacotherapy within 60 days | 93.8 | 65.0 | 100.0 | 0.0 | | |
| Psychotherapy or pharmacotherapy at all 5 timepoints^a^ | 0.0 | 0.0 | 71.2 | 0.0 | | |
| Minimally adequate psychotherapy or pharmacotherapy^b,d^ | 27.9 | 49.1 | 94.6 | 0.0 | | |
| ^a^ During the observed 150-day follow-up period  ^b^ During the first 84 days following the index depression claim, consistent with the lower bound of AACAP guidelines for psychotherapy  ^c^ Consistent with Cummings et al (2019) and the upper bound of AACAP guidelines for psychotherapy  ^d^ Consistent with our data, this was constructed as having 3 or more antidepressant prescriptions across the 5 timepoints (approximating 90 days of antidepressant prescription out of 150 days) | | | | |  |  |

| **Table S8**. Demographic and diagnostic characteristics of autistic Medicaid enrollees ages 8-29 with at least 7 out of 8 months enrollment, with a new major depressive disorder claim between 2016 and 2019 | | | | |
| --- | --- | --- | --- | --- |
|  | **Analytic sample**  (n = 44,074) | | **Disenrollment sample**  (n = 49,996) | |
| **Age,** median (IQR) | 16 (13, 20) | | 16 (13, 21) | |
| **Intellectual Disability** | **n** | **%** | **n** | **%** |
| Yes | 10,632 | 24.1 | 11,903 | 23.8 |
| No | 33,442 | 75.9 | 38,093 | 76.2 |
| **Sex** |  |  |  |  |
| Female | 13,267 | 30.1 | 15,079 | 30.2 |
| Male | 30,807 | 69.9 | 34,917 | 69.8 |
| **Race and ethnicity** |  |  |  |  |
| American Indian and Alaska Native | 561 | 1.3 | 648 | 1.3 |
| Asian/Hawaiian/Pacific Islander | 724 | 1.6 | 829 | 1.7 |
| Black | 6,508 | 14.8 | 7,347 | 14.7 |
| Hispanic/Latino | 7,664 | 17.4 | 8,641 | 17.3 |
| Multiracial | 344 | 0.8 | 387 | 0.8 |
| White | 23,901 | 54.2 | 27,169 | 54.3 |
| Missing | 4,372 | 9.9 | 4,975 | 10.0 |
| **Eligibility Group** |  |  |  |  |
| Poverty | 9,783 | 22.2 | 11,346 | 22.7 |
| Disability | 20,871 | 47.4 | 23,447 | 46.9 |
| Other^a^ | 13,420 | 30.4 | 15,203 | 30.4 |
| **Urbanicity** |  |  |  |  |
| Isolated Small Rural Town | 1,281 | 2.9 | 1,440 | 2.9 |
| Small Rural Town | 2,098 | 4.8 | 2,394 | 4.8 |
| Large Rural City/Town | 4,290 | 9.7 | 4,855 | 9.7 |
| Urban | 33,643 | 76.3 | 38,100 | 76.2 |
| Missing | 2,762 | 6.3 | 3,207 | 6.4 |
| **Any psychotherapy visit^b^** | 21,015 | 47.7 | 23,774 | 47.6 |
| **Any antidepressant prescription^b^** | 14,585 | 33.1 | 16,563 | 33.1 |
| **Any psychotherapy or antidepressant prescription^b^** | 28,074 | 63.7 | 31,775 | 63.6 |
| ^a^ Medicaid TAF data include 76 total eligibility codes. Those not linked directly to poverty or disability were combined into this category and include groups such as: those with incomes > 133% federal poverty line and under 65, expansion groups, and pregnant women  ^b^ During the observed 150-day follow-up period | | | | |

| **Table S9**. Proportion receiving psychotherapy only, pharmacotherapy only, or either during the five months following their index major depressive disorder claim among autistic Medicaid-enrollees 8-29 years old, during 2016-2019 | | | | |
| --- | --- | --- | --- | --- |
|  | **Analytic sample**  (n = 44,074) | | **Disenrollment sample**  (n = 49,996) | |
| ***Psychotherapy only*^a^** | **n** | **%** | **n** | **%** |
| Any psychotherapy visit^a^ | 21,015 | 47.7 | 23,774 | 47.6 |
| First Psychotherapy within 30 days | 14,278 | 32.4 | 16,149 | 32.3 |
| First Psychotherapy within 60 days | 17,600 | 39.9 | 19,924 | 39.9 |
| Psychotherapy visit at all 5 timepoints^a^ | 5,148 | 11.7 | 5,835 | 11.7 |
| Minimally adequate psychotherapy (> 4 visits)^b^ | 10,539 | 23.9 | 11,985 | 24.0 |
| > 8 psychotherapy visits^c^ | 4,684 | 10.6 | 5,339 | 10.7 |
| ***Pharmacotherapy only*** |  |  |  |  |
| Any antidepressant medication prescription^a^ | 14,585 | 33.1 | 16,563 | 33.1 |
| First antidepressant within 30 days | 8,100 | 18.4 | 9,133 | 18.3 |
| First antidepressant within 60 days | 10,966 | 24.9 | 12,413 | 24.8 |
| Antidepressant prescription at all 5 timepoints^a^ | 2,256 | 5.1 | 2,530 | 5.1 |
| Minimally adequate pharmacotherapy^d^ | 7,938 | 18.0 | 8,980 | 18.0 |
| ***Either psycho- or pharmacotherapy*** |  |  |  |  |
| Any psychotherapy or pharmacotherapy^a^ | 28,074 | 63.7 | 31,775 | 63.6 |
| First psychotherapy or pharmacotherapy within 30 days | 19,581 | 44.4 | 22,116 | 44.2 |
| First psychotherapy or pharmacotherapy within 60 days | 23,860 | 54.1 | 26,998 | 54.0 |
| Psychotherapy or pharmacotherapy at all 5 timepoints^a^ | 7,972 | 18.1 | 8,981 | 18.0 |
| Minimally adequate psychotherapy or pharmacotherapy^b,d^ | 16,201 | 36.8 | 18,356 | 36.7 |
| ^a^ During the observed 150-day follow-up period  ^b^ During the first 84 days following the index depression claim, consistent with the lower bound of AACAP guidelines for psychotherapy  ^c^ Consistent with Cummings et al (2019) and the upper bound of AACAP guidelines for psychotherapy  ^d^ Consistent with our data, this was constructed as having 3 or more antidepressant prescriptions across the 5 timepoints (approximating 90 days of antidepressant prescription out of 150 days) | | | | |

| **Figure S4.** Testing psychotherapy trajectory groups from 1 through 6 | |
| --- | --- |
| 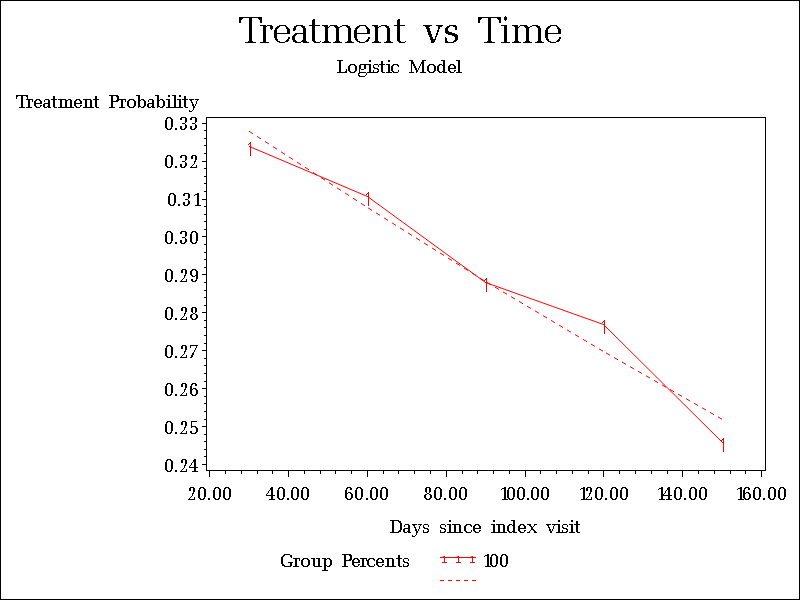 | 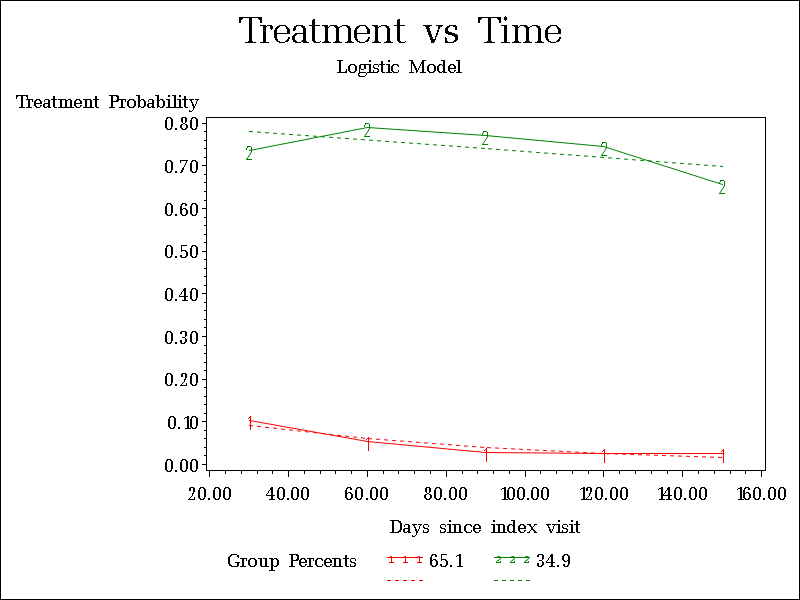 |
| 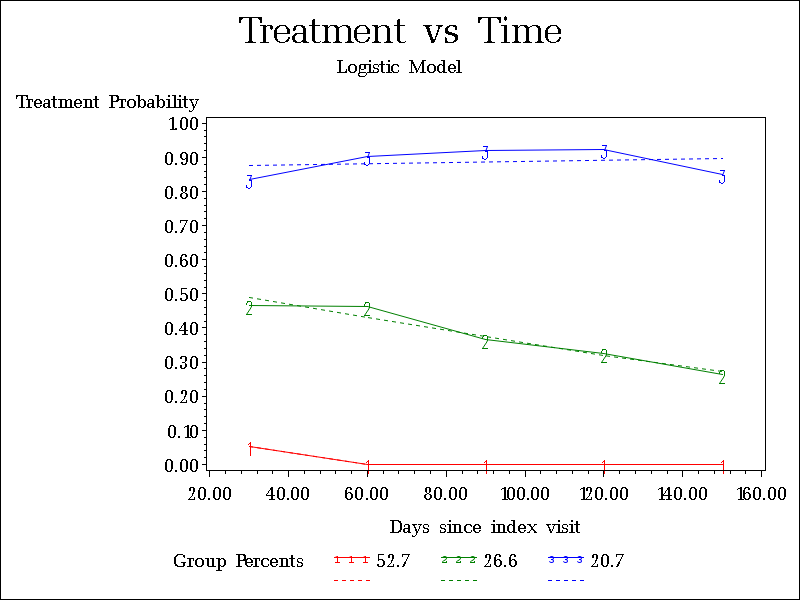 | 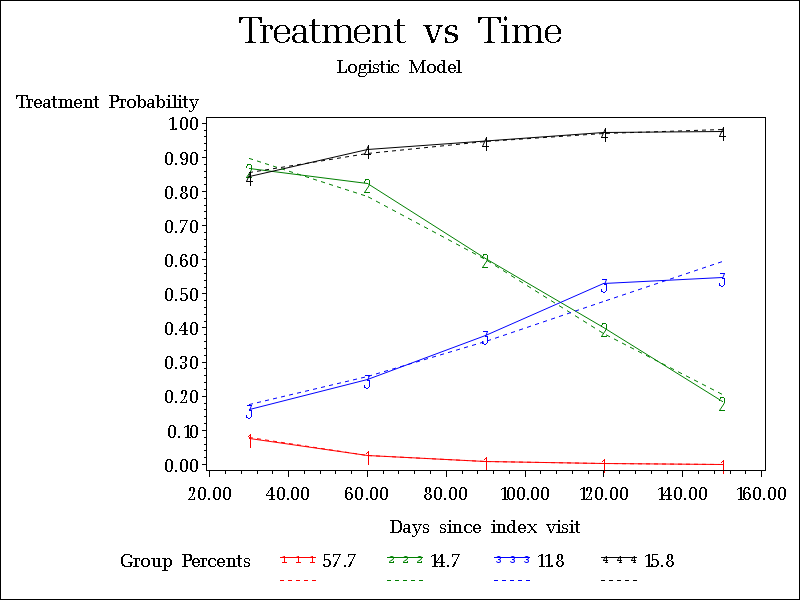 |
| 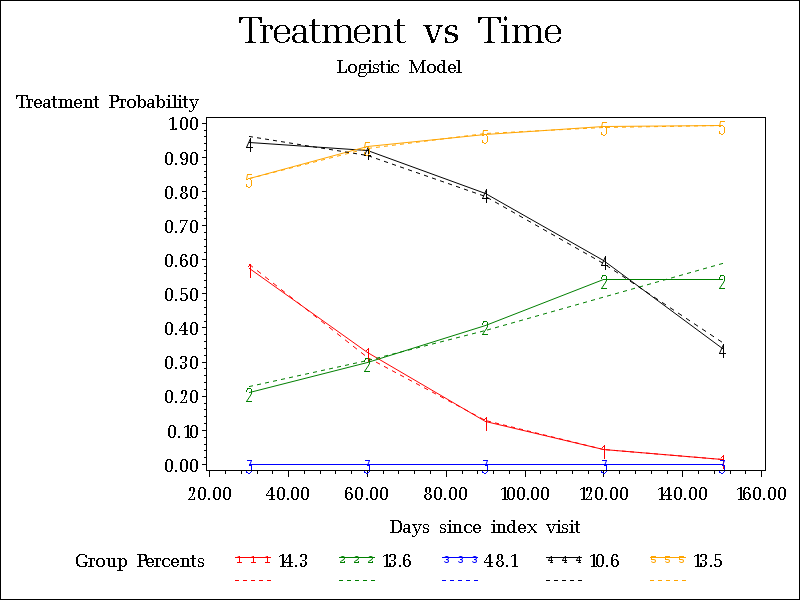 | 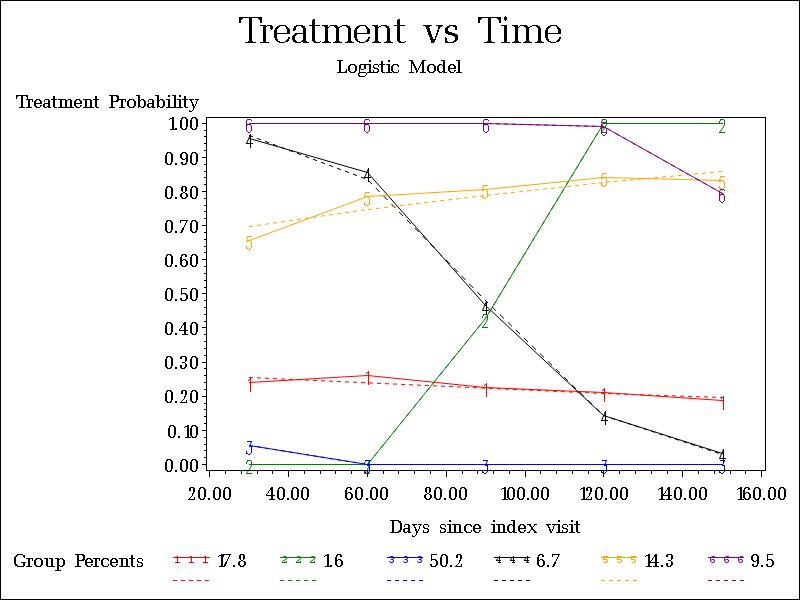 |

| **Figure S5.** Testing pharmacotherapy trajectory groups from 1 through 6 | |
| --- | --- |
| 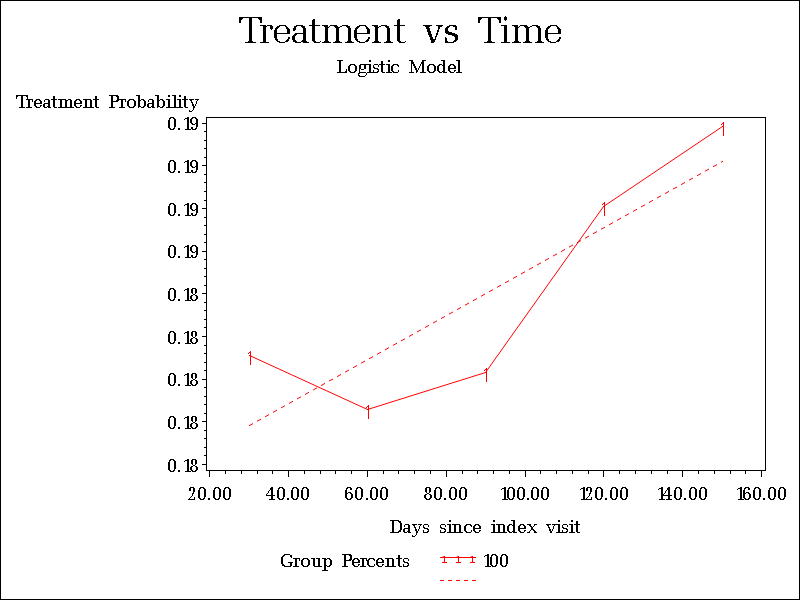 | 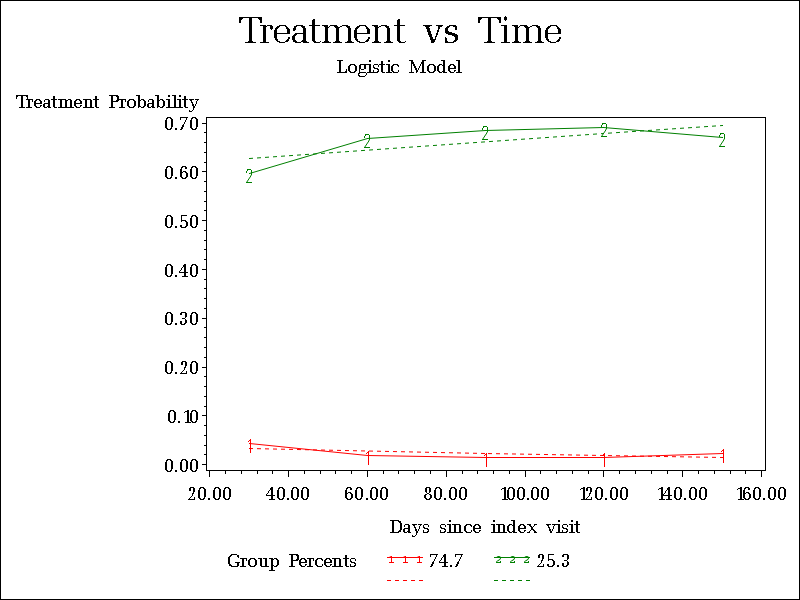 |
| 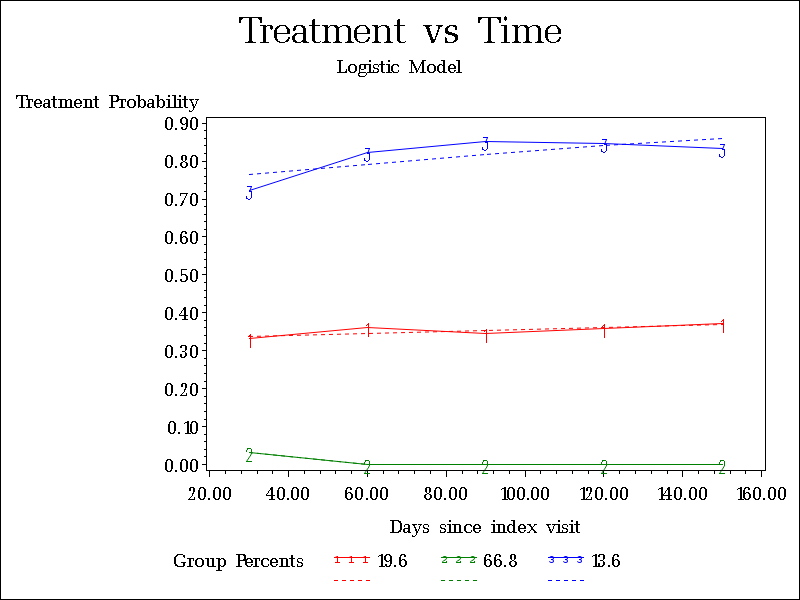 | 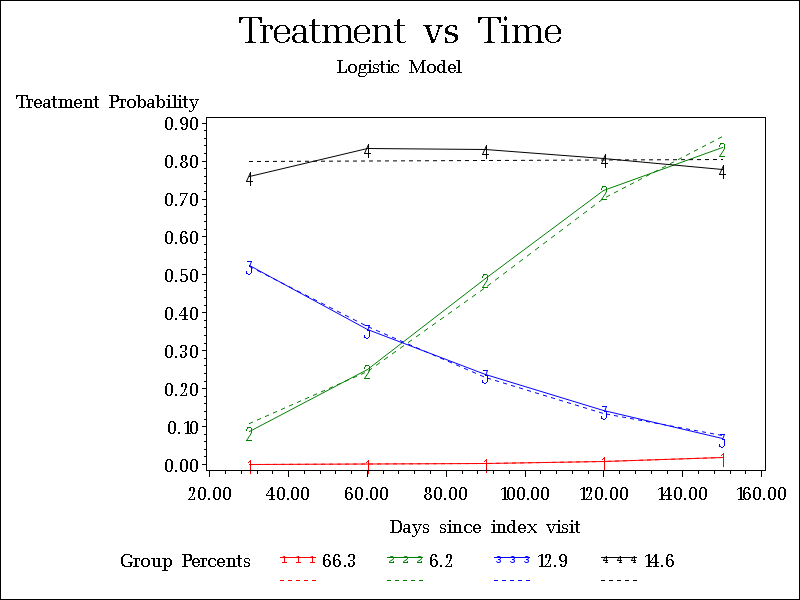 |
| 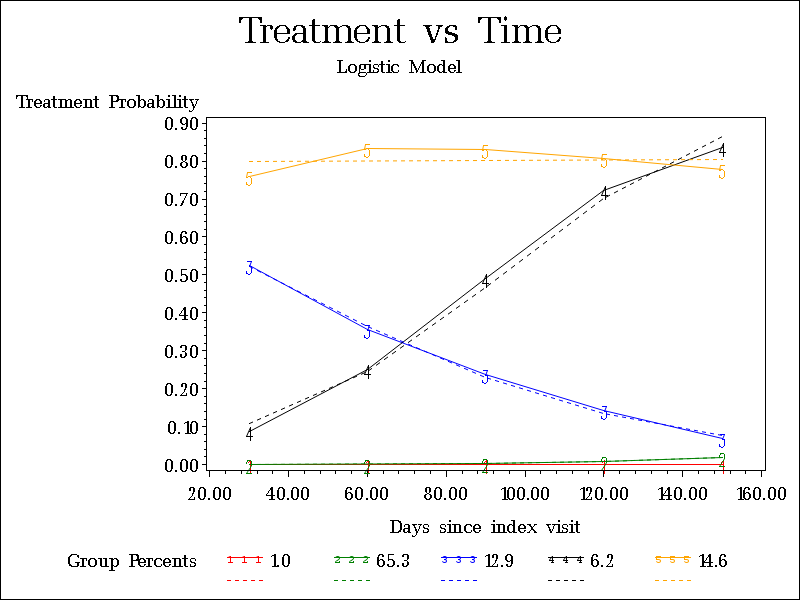 | 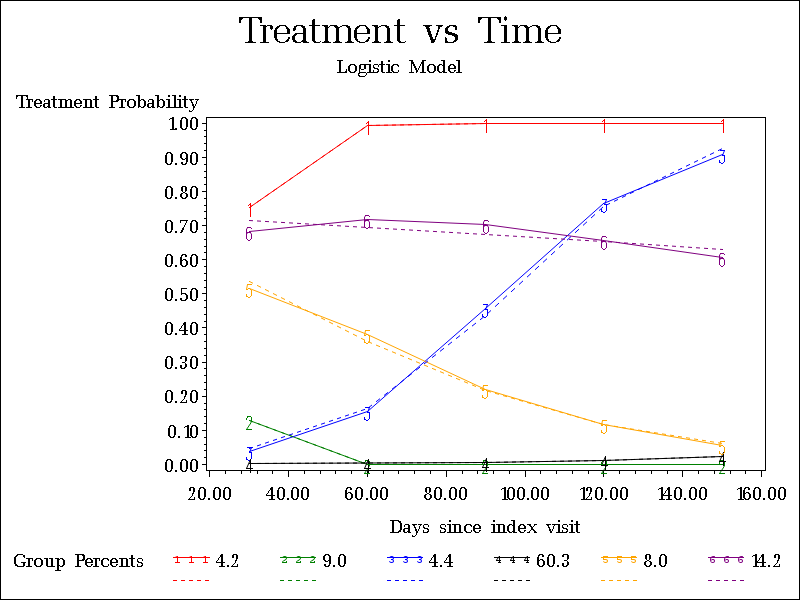 |
